# Racial Inequality in the Prevalence of Symptom-Based Depression* Versus* Self-Reported Medical Diagnosis in Brazil

**DOI:** 10.1007/s40615-025-02397-7

**Published:** 2025-04-14

**Authors:** Thais Cristina Marquezine Caldeira, Luiza Eunice Sá da Silva, Rafael Moreira Claro, Jorginete de Jesus Damião, Daniela Silva Canella, Taciana Maia de Sousa

**Affiliations:** 1 Postgraduate Program in Epidemiology, Federal University of Pelotas, Pelotas, RS Brazil; 2 Nutrition Department, Federal University of Minas Gerais, Belo Horizonte, MG Brazil; 3 Social Nutrition Department, Rio de Janeiro State University, Rio de Janeiro, RJ Brazil; 4 Applied Nutrition Department, Janeiro State University, Rio de Janeiro, RJ Brazil; 5 Postgraduate Program in Public Health, Federal University of Minas Gerais, Av. Prof. Alfredo Balena, 190, Santa Efigênia, Belo Horizonte, MG 19030130-100 Brazil

**Keywords:** Depression, Health inequities, Black people, Racial groups, Health surveys

## Abstract

A cross-sectional study was conducted with Brazilian adults from the 2019 Pesquisa Nacional de Saúde (PNS 2019; National Health Survey, in English) who self-declared race/skin color white or black (*n* = 87,187), to investigate racial inequality in the prevalence of symptom-based *versus* self-reported depression. Symptom-based depression was screened using the Patient Health Questionnaire-9 (cutoff ≥ 10), while medical diagnosis was self-reported. Prevalence of agreement (presence of medical diagnosis among those with symptoms) and disagreement (absence of medical diagnosis among those with symptoms) were compared according to race/skin color. The *Odds Ratio* (OR) for the association between both depression indicators and race/skin color were estimated by Logistic regression adjusted by socioeconomic variables. The black population had a higher prevalence of symptom-based depression than medical diagnosis (11.0% *versus* 8.6%; 2.5 percentual points (pp)), with greater differences among younger adults (6.0 pp), without a partner (3.6 pp), with lower income (3.9 pp), education (3.0 pp), less developed regions (north: 3.6 pp; northeast: 4.2 pp) and those with recent access to medical care (4.5 pp). The white population had a higher prevalence of medical diagnosis than symptom-based depression (10.6% *versus* 12.5%; -1.9 pp), with greater differences between those with higher income (-7.4 pp), education (-4.5 pp) and from south region (-6.0 pp). Disagreement was higher among black individuals while agreement was higher among white, with significant inequality among women for both agreement (33.5% in black *versus* 41.7% in white) and disagreement (66.5% in black *versus* 58.3% in white). The black population had a lower chance of having medical diagnosis of depression (OR 0.77; 95%CI 0.70–0.84) than white, regardless of socioeconomic variables. Racial inequality was identified in the prevalence of symptom-based *versus* self-reported depression, with greater inequality among women and those with lower income and education.

**Supplementary Information:**

The online version contains supplementary material available at 10.1007/s40615-025-02397-7.

## Introduction

Mental disorders are one of the main contributors to the global disease burden [1], constituting a significant public health challenge worldwide. Depression, as part of this group of illnesses, is noteworthy for its increasing prevalence, which has risen from 170.8 million cases in 1990 to 279.6 million cases in 2019 worldwide [1].

Public health interventions at the population level require continuous surveillance of the health conditions and impact of diseases on the population and healthcare systems [2, 3]. While it may not be feasible to diagnose depression through population surveys, gathering information on previous diagnoses provided by healthcare professionals or collecting data on subjective symptoms, such as alterations in thoughts, behaviors, and mood, using validated screening tools, can contribute to the assessment of the panorama of depression in a population [2, 4].

The use of self-reported information is an important strategy for epidemiological surveillance [5]. Therefore, the collection of information on self-reported medical diagnosis of depression is already well established in health surveys, such as the Behavioral Risk Factor Surveillance System (BRFSS) in the United States [2] and Surveillance System of Risk and Protection Factors for Chronic Diseases by Telephone Survey (Vigitel) [6] and the 2019 Pesquisa Nacional de Saúde (PNS 2019; National Health Survey, in English) in Brazil [7].

The use of the Patient Health Questionnaire-9 (PHQ9) scale, for example, is a validated method for collecting information about depression at a population level [2, 8]. The PHQ9 tracks the presence of nine components of the “Diagnostic and Statistical Manual of Mental Disorders, Fourth Edition, Text Revision (DSM-IV-TR)”, based on symptoms observed by a healthcare professional or reported by the patient, making it possible to identify depressive disorders larger and clinically significant (especially for a PHQ-9 ≥ 10) [2, 8].

The investigation of depression at a population level in Brazil has been carried out in 2013 and 2019 by the PNS [7]. According to this survey, the prevalence of self-reported medical diagnosis of depression in the adult population increased in this period from 7.6% to 10.2%, with a greater increase in the period among women, young people, with higher incomes and education [9]. The importance of considering social determinants when examining mental health has been reported by some studies [4, 8–10], highlighting that socioeconomic disparities may lead to an underestimation of the prevalence of depression in vulnerable populations, especially in the context of Brazil [4, 9]. One of the key factors influencing medical diagnoses of depression is the access to healthcare services, as individuals with limited access to medical care are less likely to receive a formal diagnosis, despite experiencing depressive symptoms [11]. This dependence on healthcare access can lead to disparities in reported prevalences, particularly among groups facing socioeconomic disadvantages, where barriers such as affordability, geographic distribution of services, and cultural stigmas may prevent diagnosis and treatment [11].

A worse scenario can still be observed among the black population (individuals who self-declared race/skin color black or brown). These individuals experience structural and institutional racism [12], worse living conditions, with lower access to several basic social policies, such as basic sanitation, education and health [13]. This scenario is further worsened by the presence of lower income [13]. Although previous studies have sheds light on the impact of social inequalities on mental health, it is necessary to advance the analysis by investigating the role of racial and gender in the diagnosis of depression in Brazil.

Our hypothesis is that racial inequalities influence the prevalence of self-reported medical diagnoses of depression in Brazil, with the Black population being less likely to receive a diagnosis due to structural barriers in healthcare access. We also hypothesize that these disparities differ by sex, with women experiencing a greater gap in diagnosis due to the intersection of racial and gender-related inequalities. In this sense, considering the social determinants associated with access to health and consequently with diagnosis, our objective was to investigate racial inequality in the prevalence of symptom-based versus self-reported depression in Brazil. Additional analyses stratified by sex were also conducted.

## Methods

### Design and Population Sample

We conducted a cross-sectional study with data collected by the 2019 PNS, a national population-based survey conducted by the Brazilian Institute of Geography and Statistics (IBGE) in partnership with the Ministry of Health (MoH) with the objective of producing data on the population’s health and living conditions [7, 14].

The sample is composed of permanent residents of Brazilian households based on the master sample for the IBGE’s integrated household survey system. A cluster sampling strategy (in three stages) was employed. The census tracts (organized in geographic and economic strata) were the primary sampling units. Households were second-stage units, and residents aged 15 and over were randomly selected for the interview in the third stage [7, 14]. More details on the methodology are available in specific publication [7, 14].

The present study used a subsample of 2019 PNS data composed exclusively of adult individuals (≥ 18 years of age) who declared themselves as black (including black and brown) or white (*n* = 87,187).

### Data Organization

Depression was investigated using two methods, the self-reported medical diagnosis of depression and symptom-based depression. Self-reported depression was assessed using the question: Has any doctor or mental health professional (such as a psychiatrist or psychologist) ever given you a diagnosis of depression? (yes; no). Additionally, symptom-based depression was evaluated using PHQ9 within the PNS framework [7]. The PHQ9 comprises nine questions that gauge the frequency of depressive symptoms over the past two weeks [8]. It identifies major depression based on symptoms: depressed mood, anhedonia, sleep disturbances, fatigue, appetite or weight changes, feelings of guilt or worthlessness, concentration difficulties, psychomotor changes, and thoughts of self-harm. Scores of each question ranged, according to the frequency of the symptom, from zero (no at all = 0) to (nearly every day = 3), yielding a maximum total score of 27. The cumulative score categorizes individuals into groups: without depression (0 to 4), mild depression (5 to 9), moderate depression (10 to 14), moderately severe depression (15 to 19) and severe depression (20 to 27) (Levis; Benedetti; Thombs, 2019). In this study, a score of ten points or higher (cutoff ≥ 10) indicated the presence of major depression, due to the optimized sensitivity and specificity of the test [8].

To investigate the inequalities associated with race/skin color, the official classification given by IBGE in Brazil was used, according to the self-declared about race/skin color. In Brazil, the classification of race/color/ethnicity is historically and socioculturally complex, reflecting the country’s colonial history, patterns of miscegenation, and racial inequalities. In this sense, understanding the construction of this characteristic is essential for a better understanding of the results [15]. The official categories used by the IBGE in Brazil — white, black (Preto, in Portuguese), brown (Pardo, in Portuguese), indigenous, or Asian descendants (*Amarelo,* in Portuguese)— are self-reported and represent a combination of phenotypic, cultural, and historical elements. [16]. The *Amarelo* category refers to individuals of East Asian descent, while Indigenous includes the diverse indigenous peoples of Brazil, who often face challenges of self-identification due to historical erasure and the impacts of colonization [16]. The category *Pardo*, for example, covers individuals of mixed racial heritage. Although this category has been questioned about its ability to distinguish disparities faced by populations of African descent, when disaggregated from black (*Preto*, in Portuguese), there is evidence of negative social indicators and social discrimination [16]. This indicates that people identified as brown have phenotypic traits, associated with racial marks, which expose them to the experience of racism. Therefore, the study of the black population – combining individuals who declare themselves black and brown – has been recommended and implemented in Brazil [17, 18].

Considering the context presented above, in this study we analyzed the black population (including black and brown, as described by the Brazilian Racial Equality Statute [17] and adopted by the National Policy for the Comprehensive Health of the Black Population [18] *versus* the white population. Currently, black and white populations combined represent the most prevalent racial strata in the country (99%) [19].

A set of sociodemographic characteristics complemented the analysis, such as sex (male; female), age group (18 to 24; 25 to 34; 35 to 44; 45 to 54; 55 to 64; 65 years or more), years of schooling (0 to 8 years; 9 to 11 years; and ≥ 12), *per capita* income ((< 1 minimum wage (MW); > 1 MW to < 3 MW; > 3 to < 5 MW > 5 MW), presence of partner/spouse (living with a partner; no partner), geographic region (North, Northeast, Southeast, South, Central-West) and participants who reported receiving medical care in the last 15 days at the time of the PNS survey.

### Statistical Analysis

Initially, we describe the characteristics of the studied population according to race/skin color in terms of their prevalence (%) and 95% Confidence Interval (95%CI) based on sociodemographic characteristics (sex, age group, years of schooling, per capita income, presence of partner/spouse, geographic region and medical care in the last 15 days). Subsequently, prevalence (%) of symptom-based and self-reported depression according to race/skin color were described based on sociodemographic characteristics. Differences in estimated prevalence were identified through the absolute difference in percentual points (pp) and relative difference (in percentage) between both depression indicators. Differences were identified according to the overlapping 95%CI. Additionally, we also assessed those individuals who reported receiving medical care in the last 15 days for a more in-depth evaluation of both symptomatic and self-reported depression groups (Supplementary Table 1).

We compared the percentage of agreement and disagreement between symptomatic and self-reported depression according to race/skin color, for the total population and by sex, using Equiplot. Differences were identified according to overlapping 95%CI. Agreement was considered in the presence of self-reported medical diagnosis of depression among individuals with symptoms, while disagreement was considered in the absence of a medical diagnosis among those with symptoms.

Additionally, the association between both depression indicators and race/skin color were estimated by Logistic regression models, estimating the crude and adjusted *Odds Ratios* (OR) for the total population and stratified by sex. Adjustment variables included sex (only when considered the total population), age group, education level, *per capita* income, presence of partner/spouse, geographic region and medical care in the last 15 days.

Significance level was set at 5%. Analyses and graphics were performed with Stata version 16.1 (StataCorp LP, College Station, USA), using the survey module for complex samples and significance level of 5%.

The 2019 PNS was approved by National Committee of Ethics in Research on Human Beings Conep/MoH (under opinion No 3.529.376) and the data are available for public access and use on the official IBGE website (https://www.ibge.gov.br/estatisticas/sociais/saude.html; Accessed in August 2024).

## Results

A total of 87,187 self-declared black or white Brazilian adults were investigated. The black population was younger, while the white population presented a higher education level (with double the prevalence of 12 or more years of education than the black) and higher *per capita* income (with the majority presenting 1 or more MW, while the majority of black with less than 1 MW). The North, Northeast, and Central-West regions had a higher proportion of black population, whereas the Southeast and South were predominantly white. Access to medical care in the past 15 days was more frequent among the white population (Table 1).

**Table 1:** Characteristics of the studied population according to race/skin color. Pesquisa Nacional de Saúde (National Health Survey), Brazil, 2019

Characteristics	Black				White			
	%	95%CI			%	95%CI		
Sex								
Men	47.4	46.6	-	48.2	46.0	45.1	-	46.9
Women	52.6	51.8	-	53.4	54.0	53.1	-	54.9
Age (years)								
18–24	15.3	14.6	-	15.9	12.1	11.4	-	13.0
25–34	19.3	18.7	-	19.9	16.6	15.8	-	17.3
35–44	21.4	20.8	-	22.0	18.8	18.1	-	19.5
45–54	17.6	17.0	-	18.2	18.3	17.5	-	19.0
55–64	13.8	13.4	-	14.3	16.6	15.9	-	17.2
≥ 65	12.6	12.1	-	13.1	17.7	17.0	-	18.4
Schooling (years)								
0–8	55.5	54.7	-	56.4	41.5	40.3	-	42.7
9–11	34.3	33.6	-	35.1	35.7	34.6	-	36.7
≥ 12	10.1	9.6	-	10.6	22.9	21.8	-	24.0
Per capita income (MW)								
< 1	58.7	57.9	-	59.5	33.1	31.9	-	34.3
1–2	35.7	34.9	-	36.5	47.9	46.9	-	49.0
3–5	3.5	3.2	-	3.8	10.0	9.4	-	10.6
> 5	2.1	1.9	-	2.3	9.0	8.3	-	9.7
Partner/Spouse								
No	39.1	38.2	-	39.9	37.9	37.0	-	38.9
Yes	60.9	60.1	-	61.8	62.1	61.1	-	63.0
Geographic region								
North	11.4	10.9	-	11.8	3.3	3.2	-	3.5
Northeast	35.6	34.7	-	36.4	15.0	14.5	-	15.6
Southeast	37.4	36.3	-	38.6	50.9	49.8	-	51.9
South	7.0	6.5	-	7.5	24.6	23.8	-	25.4
Central-West	8.6	8.2	-	9.0	6.2	5.9	-	6.5
Access to medical care in the past 15 days	17.5	16.9	-	18.1	19.7	19.0	-	20.5

*95%CI* 95% confidential interval, *MW minimum wage* (≈ BRL$997≈ US$274 in 2019)

The black population had a higher prevalence of symptom-based depression compared to self-reported medical diagnosis, for most of the sociodemographic groups, especially among women (15.6% *vs.* 12.6%; dif.: 3.0 pp), those aged 18 to 24 years (10.9% *vs.* 4.9%; dif.: 6.0 pp) and 25 to 34 years old (9.5% *vs.* 6.3%; dif.: 3.3 pp), with lower education level (0 to 8 years: 12.1% *vs.* 9.1%; dif.: 3.0 pp, and 9 to 11 years: 10.0% *vs*. 7.3%; dif.: 2.6 pp), *per capita* income lower than 1 MW (11.9% *vs*. 8.0%; dif.: 3.9 pp),without a partner (13.0% *vs.* 9.4%; dif.: 3.6 pp), from the North (8.3% vs. 4.7%; dif.: 3.6 pp) and Northeast (10.9% vs. 6.6%; dif.: 4.2 pp) region and access to medical care in the past 15 days (19.6% vs. 15.1%; dif.: 4.5 pp). The opposite scenario was observed among the self-declared white, where most sociodemographic groups presented a higher prevalence of self-reported medical diagnosis compared to symptom-based depression, especially among women (14.3% *vs.* 17.5%; dif.: −3.2 pp), adults over 45 years of age, with higher education level (9.2% *vs.* 13.7%; dif.: −4.5 pp), higher *per capita* income, among adults with a partner (9.3% *vs.* 11.7%; dif.: −2.4 pp), from the southern region (9.8% vs. 15.8%; dif.: −6.0 pp) and access to medical care in the past 15 days (17.3% vs. 20.5%; dif.: −3.3 pp). The only exception was among the younger population (aged 18 to 24 years) (Table 2). A similar discrepancy, although more pronounced for some sociodemographic groups, was identified considering the subsample of individuals who had medical care in the last 15 days (Supplementary Table 1).

**Table 2:** Prevalence (%) of symptom-based and self-reported depression according to race/skin color. Pesquisa Nacional de Saúde (National Health Survey), Brazil, 2019

Group	Black											White								
	Symptom-based				Self-reported diagnosis				dif			Symptom-based			Self-reported diagnosis				dif	
	%	95%CI			%	95%CI			pp	%	%	95%CI			%	95%CI			pp	%
Sex																				
Men	6.0	5.4	-	6.6	4.1	3.6	-	4.6	**1.9**	**31.6**	6.2	5.6	-	6.9	6.5	5.9	-	7.2	−0.3	−5.2
Women	15.6	14.8	-	16.3	12.6	11.9	-	13.3	**3.0**	**19.3**	14.3	13.4	-	15.3	17.5	16.5	-	18.6	**−3.2**	**−22.5**
Age																				
18–24	10.9	9.3	-	12.7	4.9	3.8	-	6.3	**6.0**	**54.9**	11.2	9.2	-	13.5	7.3	5.7	-	9.3	**3.9**	**34.5**
25–34	9.5	8.7	-	10.4	6.3	5.5	-	7.1	**3.3**	**34.3**	9.7	8.1	-	11.5	8.4	7.2	-	9.8	1.3	13.0
35–44	11.0	10.1	-	12.0	9.4	8.5	-	10.4	1.6	14.6	10.1	8.9	-	11.4	13.0	11.3	-	14.8	−2.9	−28.4
45–54	12.5	11.4	-	13.8	11.0	10.0	-	12.1	1.5	12.2	10.9	9.7	-	12.3	14.9	13.3	-	16.6	**−4.0**	**−36.3**
55–64	11.7	10.6	-	13.0	11.5	10.4	-	12.7	0.3	2.1	10.8	9.4	-	12.4	15.5	13.7	-	17.4	**−4.7**	**−43.4**
≥ 65	10.8	9.7	-	12.0	8.4	7.5	-	9.5	**2.4**	**21.8**	11.0	9.7	-	12.3	13.9	12.6	-	15.4	**−3.0**	**−26.9**
Schooling																				
0–8	12.1	11.5	-	12.7	9.1	8.5	-	9.7	**3.0**	**25.0**	12.2	11.2	-	13.1	12.9	12.0	-	13.8	−0.7	−6.0
9a11	10.0	9.2	-	10.8	7.3	6.6	-	8.1	**2.6**	**26.3**	9.7	8.7	-	10.7	11.2	10.1	-	12.3	−1.5	−15.6
≥ 12	9.0	7.9	-	10.2	9.9	8.6	-	11.3	−0.9	−10.2	9.2	7.8	-	10.7	13.7	12.4	-	15.1	**−4.5**	**−49.7**
Per capita income																				
< 1	11.9	11.3	-	12.6	8.0	7.4	-	8.6	**3.9**	**33.0**	13.1	12.0	-	14.2	11.5	10.4	-	12.8	1.5	11.7
1a2	10.0	9.3	-	10.8	9.3	8.5	-	10.1	0.8	7.5	10.1	9.2	-	11.1	12.5	11.6	-	13.5	**−2.4**	**−23.6**
3a5	8.0	6.4	-	10.0	9.8	8.2	-	11.8	−1.8	−22.1	7.5	6.0	-	9.3	13.3	11.5	-	15.3	**−5.8**	**−78.0**
> 5	9.7	7.1	-	13.1	10.7	8.3	-	13.8	−1.1	−11.3	7.4	6.1	-	9.0	14.8	13.0	-	16.7	**−7.4**	**−99.4**
Partner/Spouse																				
No	13.0	12.2	-	13.9	9.4	8.7	-	10.2	**3.6**	**27.7**	12.7	11.6	-	13.8	13.7	12.7	-	14.8	−1.0	−8.2
Yes	9.8	9.2	-	10.3	8.0	7.5	-	8.6	**1.8**	**18.0**	9.3	8.6	-	10.0	11.7	10.9	-	12.5	**−2.4**	**−25.8**
Geographic region																				
North	8.3	7.5	-	9.1	4.7	4.0	-	5.4	**3.6**	**43.7**	8.0	6.6	-	9.4	6.1	5.0	-	7.3	**1.9**	**23.3**
Northeast	10.9	10.2	-	11.5	6.6	6.1	-	7.1	**4.2**	**39.0**	10.1	9.1	-	11.0	7.8	6.9	-	8.6	**2.3**	**23.1**
Southeast	11.8	10.8	-	12.8	10.3	9.3	-	11.2	**1.5**	**12.7**	11.2	10.1	-	12.3	12.8	11.7	-	13.9	**−1.5**	**−13.6**
South	11.8	10.0	-	13.6	14.1	12.2	-	16.0	**−2.3**	**−19.5**	9.8	8.9	-	10.7	15.8	14.7	-	16.9	**−6.0**	**−61.6**
Central-West	11.6	10.4	-	12.8	9.7	8.5	-	10.9	**1.9**	**16.6**	10.9	9.1	-	12.7	11.5	10.0	-	13.0	**−0.6**	**−5.3**
Access to medical care in the past 15 days	19.6	18.08	-	21.1	15.1	13.7	-	16.5	**4.5**	**23.1**	17.3	15.7	-	18.1	20.5	18.8	-	22.2	**−3.3**	**−18.8**
Total	11.0	10.6	-	11.5	8.6	8.1	-	9.0	**2.5**	**22.5**	10.6	10.0	-	11.2	12.5	11.8	-	13.1	**−1.9**	**−17.8**

Significative differences according to overlap of 95% confidence intervals are highlighted in bold. dif.: difference between the prevalence of symptom-based and self-reported depression in percentual points (pp.) and relative percentual (%), using symptom-based prevalence as reference value; *95%CI* 95% confidence interval, *MW* minimum wage (≈ BRL$997≈ US$274 in 2019)

**Fig. 1:**
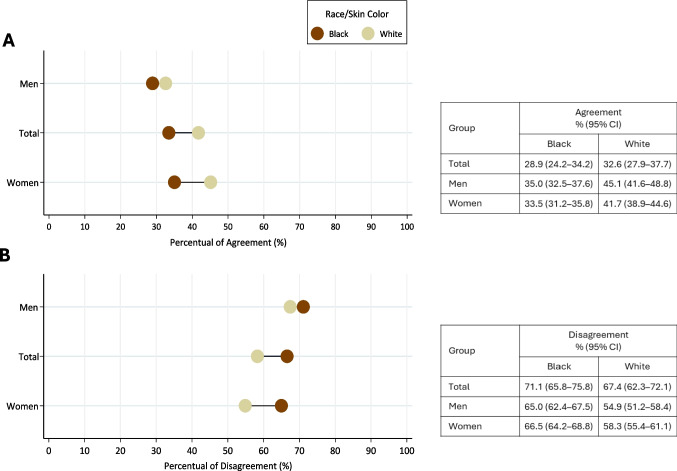
Percentual (%) of agreement and disagreement between symptom-based and self-reported depression according to race/skin color for the total population and by sex. Pesquisa Nacional de Saúde (National Health Survey), Brazil, 2019. Note: **A **Agreement (Presence of medical diagnosis of depression among those with symptoms). **B** Disagreement (Absence of medical diagnosis among those with symptoms)

Overall, the prevalence of disagreement (absence of a medical diagnosis of depression among those with symptoms) was higher among the black population, while the prevalence of agreement (presence of a medical diagnosis of depression among those with symptoms) was higher in the white population. This inequality was significant among women for both agreement (33.5% among black *vs.* 41.7% among white) and disagreement (66.5% among black *vs.* 58.3% among white) (Fig. 1).

According to the analyses adjusted by sociodemographic characteristics, the black population was less likely to have received a medical diagnosis of depression (OR: 0.77; 95%CI 0.70–0.84) than the white population. A similar result was found for the models stratified according to sex, with a slightly lower chance to have received a medical diagnosis of depression among black men (OR: 0.74; 95%CI 0.62–0.88) than black women (OR: 0.78; 95%CI 0.70–0.86). Regarding the presence of symptom-based depression, no association was found related to race/skin color (Table 3).

**Table 3:** Odds Ratio (OR) for the association between symptom-based and self-reported diagnosis of depression with race/skin color. Pesquisa Nacional de Saúde (National Health Survey), Brazil, 2019

Groups	Symptom-based								Self-reported diagnosis							
	Crude OR	95%CI			Adjusted OR	95%CI			Crude OR	95%CI			Adjusted OR	95%CI		
Total																
White	1.00				1.00				1.00				1.00			
Black	1.05	0.97	-	1.14	0.99	0.91	-	1.08	0.66**	0.61	-	0.71	0.77**	0.70	-	0.84
Men																
White	1.00				1.00				1.00				1.00			
Black	0.92	0.78	-	1.08	0.92	0.78	-	1.08	0.61**	0.52	-	0.73	0.74*	0.62	-	0.88
Women																
White	1.00				1.00				1.00				1.00			
Black	1.11	1.01	-	1.21	1.02	0.93	-	1.13	0.68**	0.62	-	0.74	0.78**	0.70	-	0.86

*OR* Odds Ratio, *95%CI* 95% confidence interval. Total: adjusted by gender, age group, education level, per capita income, and presence of partner/spouse. Men/Women: adjusted by age group, education level, per capita income, presence of partner/spouse, geographic region, and access to medical care in the past 15 days. *p-value < 0.05; **p-value < 0.001

## Discussion

Aiming to identify racial disparities in the prevalence of depression in Brazil by comparing symptom-based depression and self-reported medical diagnosis, we investigated data from 87,187 self-declared black or white Brazilian adults. The black population exhibited a higher prevalence of symptom-based depression, particularly among women and young adults. In contrast, self-declared white people had a higher prevalence of self-reported medical diagnosis, especially among older adults, those with higher educational levels and income, and those who had a medical appointment in the previous 15 days. Furthermore, a higher prevalence of depression was observed in the more developed regions (Southeast and South), while the greatest discrepancy in symptoms-based depression and a formal diagnosis was observed among black individuals in the North and Northeast regions. Adjusted analyses showed that black individuals were less likely to receive a self-reported medical diagnosis. The disagreement between symptom-based depression (yes) and self-reported medical diagnosis (no) was greater among black individuals, especially women. These findings highlight racial disparities in the perception and diagnosis of depression in Brazil.

Our results contribute to existing knowledge on depressive disorders among adults in Brazil. Several studies using data from the 2019 PNS have explored different aspects of depression prevalence and underdiagnosis. One study found a prevalence of depression based on the PHQ-9 screening (cutoff point ≥ 10) similar to our results, reporting higher prevalence among women, black individuals, those without a partner, individuals with no formal education or incomplete primary education, and those living in urban areas [20]. In contrast, another study that analyzed self-reported medical diagnoses of depression found higher prevalence among women, white individuals, those with higher education, and residents of urban areas [21]. Furthermore, a previous study estimated that 63.6% of individuals with depression detected by the PHQ-9 had not receive a formal diagnosis, with underdiagnosis being more prevalent among men, older adults, individuals with lower income and education levels, and residents of less developed regions in Brazil. However, in that study, racial disparities were not found to be significant, as expected due to this association already identified in previous studies [4].

Some important differences should be noted between the results obtained between our study and the one carried out by Faisal-Cury et al. (2022). First, they restricted analysis to individuals who received medical care in the previous 15 days, whereas we treated this variable as an adjustment factor rather than a population selection criterion. Another key difference is the PHQ-9 cutoff, while Faisal-Cury et al. (2022) used a cutoff of 9 points, we adopted a cutoff of 10 to identify moderate depression, enhancing the specificity of the score. We emphasize these differences particularly considering structural inequalities in healthcare access, which extend beyond the opportunity to attend a medical appointment [22, 23]. Factors such as the quality of care and structural racism likely play a critical role in the underdiagnosis of depression among black individuals, those living in economically disadvantaged regions (North, Northeast, and Central West) and those with lower incomes. Racial biases in medical training, healthcare providers’ perceptions, and systemic barriers may contribute to diagnostic gaps, leading to lower detection rates and inadequate health care for marginalized populations [24]. To further explore these disparities, we include a supplementary table comparing self-reported and symptom-based depression by sociodemographic characteristics among individuals who received medical care in the past 15 days. The supplementary results reinforce the patterns observed in the overall population, regardless of recent medical care.

In Latin America, regional prevalence rates of depression have been reported at 12%, significantly higher than the global average of 5%. This scenario may be associated with high socioeconomic inequality, social polarization, and distrust in institutions, all of which contribute to poor social cohesion [25, 26]. A 2018 study found that the prevalence of depressive symptoms in Peru was 6.2% in the last year of data collection, with notable differences in treatment especially among population groups, especially in rural areas and individuals with low levels of wealth [27]. Also, in a study carried out between 2010 and 2011 in cities across Argentina, Chile and Uruguay observed a depression prevalence of 14.6%, with higher rates in medium-sized urban centers [28]. More recent studies with national representativeness conducted in Latin American countries are scarce, highlighting the relevance of the present study in filling this gap in the literature.

In Brazil, despite the higher prevalence of depression in more developed areas, as observed in this study for the South and Southeast regions and in previous study analyzing PHQ-9 data in urban areas [20], the greatest disparities in diagnosis are found in less developed regions [4], particularly among the black population. Notably, variations in study methods, such as different cutoff points for categorizing depression severity, can impact prevalence estimates. While the PHQ-9 is widely used across countries [25–30], tracking self-reported medical diagnoses remains essential for identifying disparities in healthcare access among vulnerable populations.

Although socioeconomic disparities in depressive disorders are well-documented, there has been limited focus on how these estimates are collected, particularly among vulnerable populations. Racial disparities in mental health diagnoses persist as a structural issue, with marginalized groups often facing barriers to accessing mental health services and receiving adequate diagnosis and care compared to their white counterparts [31]. A survey by a U.S. health insurer between 2016 and 2020 revealed significant disparities in the diagnosis and treatment of black and Hispanic individuals. Among the stigmas reported, contempt for these communities was the most frequently cited, highlighting the influence of racism in patient-provider interactions [32].

Regarding the disagreement between symptom-based and self-reported diagnosis, 12% of black women who screened positive for major depression had not received a formal diagnosis. Depression diagnosis is influenced by the availability and access to healthcare professionals, and the black population in Brazil frequently experience lower access to health services [13]. Data from the 2019 PNS in Brazil indicated that 86.4% of white women consulted a doctor in the past year compared to 82.8% of black women [33]. Furthermore, Brazil’s severe economic and political crisis, which began in 2016, led to increased unemployment and cuts in public funding, particularly for health and education. This crisis negatively impacted service availability and exacerbated the socioeconomic conditions of vulnerable groups [34]. Combined with the effects of the COVID-19 pandemic, Brazil’s lack of effective social protection mechanisms likely intensified economic and health hardships for these groups [35–37].

The “World Health Organization’s (WHO) “Mental Health Action Plan 2013–2030” emphasizes the need to address disparities by proactively identifying and supporting groups at higher risk of mental illness with limited access to services. The plan includes updated mental health targets for 2030, aiming for comprehensive, integrated services in community-based settings, with a goal for 80% of countries to integrate mental health into primary healthcare [38].

Brazil has the Unified Health System (SUS), a national public health system that includes mental health services. Different mental health care services are integrated into the Psychosocial Care Network (RAPS), with the establishment of intersectoral actions to ensure comprehensive care [39]. However, coverage of these services remains low across various regions of Brazil, especially less developed. As response to the health inequalities affecting black population in Brazil, in 2009 the Ministry of Health published the National Policy for the Comprehensive Health of the Black Population [40]. However, even after 15 years, significant disparities persist, probably due to the policy’s limited implementation [18]. Additionally, challenges such as high turnover rates among healthcare professionals and inadequate training in addressing the mental health needs of marginalized populations remain an issue [41]. Addressing structural racism in healthcare and society requires further progress. Studies that expose these systemic inequalities and provide evidence for equitable public policies are essential [42]. A humanized approach to training healthcare professionals, particularly regarding the health care of the Black population, is a strategic measure outlined in the National Policy, though its implementation has been minimal.

Furthermore, it is necessary to highlight out our choice to translate *Negro* as "Black" to align with international academic and sociopolitical terminology. The term *Negro* in Brazil is commonly used to encompass both *Preto (black)* and *Pardo (brown)* individuals when addressing racial inequalities affecting populations of African descent. This collective use of *Negro* emphasizes shared experiences of systemic racism and social exclusion, particularly in health, education, and income disparities. Our choice to translate *Negro* as "black" reflects this sociopolitical framing, recognizing that "black" in international discourse often serves as an inclusive term for individuals of African descent, irrespective of national or cultural differences. While we acknowledge that the direct translation of *Pardo* as "brown" and *Preto* as "black" might appear more literal, such an approach could fragment the understanding of racial inequalities that the category *Negro* seeks to highlight in the Brazilian context.

This study has some limitations. Although the PNS provides valuable data on depression prevalence, it was not specifically designed to assess mental health. However, comparing different depression indicators shed light on disparities in access to medical diagnosis between the black and white populations in Brazil. Furthermore, the PNS has the potential to assess depression through two different methods, and it is recommended that both indicators be evaluated together whenever possible, with the aim of minimizing biases in symptom reporting and access to the medical diagnosis of depression. The data was collected in 2019 and considering all the changes in the social context caused by the COVID-19 pandemic [35], important changes in sociodemographic and mental health indicators may have occurred in the population. Despite the higher prevalence of symptom-based depression among the Black population, they report medical diagnosis less frequently, especially among younger individuals, and those with lower education and income levels. The lowest chance of receiving a medical diagnosis is observed among black women, regardless of other sociodemographic characteristics. Considering these findings, we recommend that public mental health policies incorporate anti-racist approaches to face eliminate racial inequities and remove barriers to mental healthcare access for the Black population.

## Supplementary Information

Below is the link to the electronic supplementary material.

ESM Supplementary Material 1 (DOCX 31.1 KB)

## Data Availability

Data from the Pesquisa Nacional de Saúde 2019 (National Health Survey 2019) are available for public access and use on the official IBGE website (https://www.ibge.gov.br/estatisticas/sociais/saude.html; Accessed in August 2024).
