# Supplementary material for: Racial Inequality in the Prevalence of Symptom-Based Depression Versus Self-Reported Medical Diagnosis in Brazil
Source: J Racial Ethn Health Disparities. 2025 Apr 14;13(3):2049–58. doi: 10.1007/s40615-025-02397-7 (PMC13157428; doi:10.1007/s40615-025-02397-7)
Supplement: Supplementary file 1 — Supplementary Material 1 (DOCX 31.1 KB) [file 40615_2025_2397_MOESM1_ESM.docx]

Supplementary

Table S1: Prevalence (%) of symptom-based and self-reported depression among individuals with a medical appointment in the last 15 days according to race/skin color. Pesquisa Nacional de Saúde (National Health Survey), Brazil, 2019.

| **Group** | **Black/Mixed race** | | | | | | | | | | **White** | | | | | | | | | |
| --- | --- | --- | --- | --- | --- | --- | --- | --- | --- | --- | --- | --- | --- | --- | --- | --- | --- | --- | --- | --- |
|  | **Symptom-based (with medical care in the last 15 days)** | | | | **Self-reported diagnosis (with medical care in the last 15 days)** | | | | **dif.** | | **Symptom-based (with medical care in the last 15 days)** | | | | **Self-reported diagnosis (with medical care in the last 15 days)** | | | | **dif.** | |
|  | **%** | **95%CI** | | | **%** | **95%CI** | | | **pp.** | **%** | **%** | **95%CI** | | | **%** | **95%CI** | | | **pp.** | **%** |
| **Sex** |  |  |  |  |  |  |  |  |  |  |  |  |  |  |  |  |  |  |  |  |
| Men | 13.2 | 10.9 | - | 16.0 | 9.0 | 7.0 | - | 11.5 | **4.3** | **32.2** | 11.0 | 9.1 | - | 13.3 | 11.2 | 9.4 | - | 13.4 | -0.2 | -1.9 |
| Women | 23.4 | 21.6 | - | 25.4 | 18.7 | 17.0 | - | 20.6 | **4.7** | **20.0** | 21.0 | 18.9 | - | 23.2 | 26.0 | 23.7 | - | 28.4 | **-5.0** | **-24.0** |
| **Age** |  |  |  |  |  |  |  |  |  |  |  |  |  |  |  |  |  |  |  |  |
| 18-24 | 10.3 | 15.1 | - | 29.0 | 13.0 | 6.6 | - | 19.4 | -2.7 | -26.4 | 15.5 | 9.5 | - | 21.5 | 10.3 | 6.1 | - | 14.5 | **5.2** | **33.5** |
| 25-34 | 14.4 | 14.2 | - | 21.0 | 13.5 | 10.3 | - | 16.7 | 0.9 | 6.4 | 14.2 | 10.3 | - | 18.1 | 14.4 | 10.6 | - | 18.3 | -0.3 | -1.8 |
| 35-44 | 22.7 | 18.4 | - | 25.0 | 16.5 | 13.4 | - | 19.7 | **6.2** | **27.2** | 16.9 | 13.1 | - | 20.6 | 22.7 | 18.5 | - | 26.9 | **-5.8** | **-34.5** |
| 45-54 | 24.0 | 17.6 | - | 24.5 | 18.0 | 14.8 | - | 21.2 | **6.0** | **24.9** | 21.9 | 17.8 | - | 26.1 | 24.0 | 19.4 | - | 28.5 | -2.0 | -9.2 |
| 55-64 | 24.7 | 17.2 | - | 23.5 | 17.8 | 14.7 | - | 21.0 | **6.9** | **27.8** | 19.3 | 15.7 | - | 22.9 | 24.7 | 19.6 | - | 29.8 | **-5.4** | **-28.2** |
| ≥65 | 20.3 | 12.5 | - | 17.6 | 10.7 | 8.6 | - | 12.8 | **9.6** | **47.3** | 14.9 | 12.2 | - | 17.5 | 20.3 | 17.1 | - | 23.6 | **-5.5** | **-36.8** |
| **Schooling** |  |  |  |  |  |  |  |  |  |  |  |  |  |  |  |  |  |  |  |  |
| 0-8 | 19.8 | 19.3 | - | 23.2 | 15.9 | 14.0 | - | 17.8 | **3.9** | **19.8** | 20.7 | 18.1 | - | 23.2 | 19.8 | 17.3 | - | 22.3 | 0.9 | 4.2 |
| 9a11 | 21.0 | 15.2 | - | 21.0 | 13.0 | 10.4 | - | 15.6 | **8.0** | **38.1** | 16.3 | 13.4 | - | 19.1 | 21.0 | 17.9 | - | 24.2 | **-4.7** | **-29.0** |
| ≥12 | 21.2 | 13.0 | - | 20.0 | 17.1 | 13.5 | - | 20.6 | **4.2** | **19.6** | 13.1 | 10.3 | - | 15.9 | 21.2 | 17.8 | - | 24.6 | **-8.1** | **-62.3** |
| **Per capita income** |  |  |  |  |  |  |  |  |  |  |  |  |  |  |  |  |  |  |  |  |
| <1 | 19.7 | 19.8 | - | 24.2 | 15.2 | 13.2 | - | 17.1 | **4.6** | **23.1** | 23.2 | 20.0 | - | 26.4 | 19.7 | 16.1 | - | 23.4 | **3.5** | **15.0** |
| 1a2 | 21.3 | 15.1 | - | 20.0 | 15.1 | 12.8 | - | 17.4 | **6.2** | **29.1** | 17.1 | 14.7 | - | 19.5 | 21.3 | 18.8 | - | 23.8 | **-4.2** | **-24.6** |
| 3a5 | 18.4 | 7.4 | - | 14.6 | 13.0 | 9.1 | - | 16.8 | **5.5** | **29.7** | 9.2 | 6.4 | - | 12.1 | 18.4 | 14.4 | - | 22.5 | **-9.2** | **-99.7** |
| >5 | 22.0 | 8.1 | - | 22.7 | 16.2 | 9.0 | - | 23.4 | **5.8** | **26.4** | 11.3 | 8.0 | - | 14.6 | 22.0 | 17.4 | - | 26.6 | **-10.7** | **-95.0** |
| **Partner/Spouse** |  |  |  |  |  |  |  |  |  |  |  |  |  |  |  |  |  |  |  |  |
| No | 22.3 | 21.0 | - | 26.7 | 18.0 | 15.4 | - | 20.7 | **4.2** | **18.9** | 17.8 | 15.4 | - | 20.3 | 22.3 | 19.5 | - | 25.0 | **-4.4** | **-24.9** |
| Yes | 19.6 | 15.4 | - | 18.8 | 13.3 | 11.7 | - | 14.9 | **6.3** | **32.0** | 17.0 | 14.9 | - | 19.0 | 19.6 | 17.3 | - | 21.8 | -2.6 | -15.2 |
| **Geographic region** |  |  |  |  |  |  |  |  |  |  |  |  |  |  |  |  |  |  |  |  |
| North | 12.0 | 15.2 | - | 21.0 | 7.7 | 5.9 | - | 9.4 | **4.3** | **35.8** | 13.2 | 9.0 | - | 17.4 | 12.0 | 7.0 | - | 16.9 | 1.2 | 9.4 |
| Northeast | 13.7 | 16.3 | - | 20.3 | 10.9 | 9.5 | - | 12.3 | 2.8 | 20.4 | 16.5 | 13.4 | - | 19.5 | 13.7 | 10.8 | - | 16.5 | 2.8 | 16.9 |
| Southeast | 20.6 | 17.0 | - | 22.8 | 17.7 | 14.9 | - | 20.5 | 2.9 | 14.2 | 17.0 | 14.4 | - | 19.6 | 20.6 | 17.9 | - | 23.4 | **-3.6** | **-21.3** |
| South | 25.4 | 18.5 | - | 27.7 | 25.1 | 19.9 | - | 30.2 | 0.3 | 1.2 | 18.4 | 15.8 | - | 21.0 | 25.4 | 22.6 | - | 28.2 | **-7.0** | **-37.9** |
| Central-West | 17.6 | 17.3 | - | 25.2 | 14.8 | 11.7 | - | 17.9 | 2.8 | 15.8 | 18.6 | 13.9 | - | 23.2 | 17.6 | 13.5 | - | 21.6 | 1.0 | 5.4 |
| **Total** | 20.5 | 18.1 | - | 21.1 | 15.1 | 13.7 | - | 16.5 | **5.5** | **26.7** | 17.3 | 15.7 | - | 18.9 | 20.5 | 18.8 | - | 22.3 | **-3.3** | **-18.8** |

**Note:** significative differences according to the overlap of 95% confidence intervals are highlighted in bold. dif.: difference between the prevalence of symptom-based and self-reported depression in percentual points (pp.) and relative percentual (%), using symptom-based prevalence as reference value; 95%CI: 95% confidence interval; MW: minimum wage (≈ BRL$997≈ US$274 in 2019).
